# The dark matter of bacterial genomic surveillance—antimicrobial resistance plasmid transmissions in the hospital setting

**DOI:** 10.1128/jcm.00121-25

**Published:** 2025-05-12

**Authors:** Annika Sobkowiak, Vera Schwierzeck, Vincent van Almsick, Natalie Scherff, Franziska Schuler, Kyrylo Bessonov, James Robertson, Dag Harmsen, Alexander Mellmann

**Affiliations:** 1 Institute of Hygiene, University Hospital Münster 39069, Münster, North Rhine-Westphalia, Germany; 2 Department of Cardiology I – Coronary and Peripheral Vascular Disease, Heart Failure, University Hospital Munster 570237, Münster, North Rhine-Westphalia, Germany; 3 Institute of Medical Microbiology, University Hospital Münster 39069, Münster, North Rhine-Westphalia, Germany; 4 National Microbiology Laboratory, Public Health Agency of Canada, Guelph, Canada; 5 Department of Periodontology and Restorative Dentistry, University Hospital Münster 39069, Münster, North Rhine-Westphalia, Germany; Maine Medical Center Department of Medicine, Portland, Maine, USA

**Keywords:** Antimicrobial resistance, AMR, plasmid, long-read whole genome sequencing, plasmid transmission, clonal transmission, prospective surveillance

## Abstract

Dissemination of antimicrobial resistance (AMR) is a growing global public health burden. The aim of this study was to characterize AMR plasmid transmissions within a tertiary care hospital and identify relevant AMR plasmid transmission pathways. During an 18-month observation period, 540 clinical gram-negative multidrug-resistant bacterial (MDRB) isolates were collected during routine hospital surveillance and subjected to Pacific Biosciences long-read whole genome sequencing. Potential clonal transmissions were determined based on core genome multilocus sequence typing (cgMLST), and plasmid transmissions were detected using a novel real-time applicable tool for plasmid transmission detection. Potential transmissions were validated using epidemiological data. Among the 471 eligible MDRB isolates, we detected 1,539 plasmids; 84.41% of these were circularized. We identified 38 potential clonal transmissions in 24 clusters based on cgMLST and 121 potential plasmid transmissions in 24 clusters containing genetically related AMR plasmids. Among the latter clusters, 10 contained different multilocus sequence types (involving 2–38 isolates, median: 3 isolates), and nine contained multiple species (2–18 isolates, median: 4). Epidemiological data confirmed 19 clonal transmissions (in seven clusters) and an additional 12 plasmid transmissions (within eight plasmid clusters). Among these, we identified seven cases of intra-host and five patient-to-patient plasmid transmissions. We demonstrate that intra-host and patient-to-patient transmissions of AMR plasmids can be identified by combining long-read sequencing with real-time applicable tools during routine molecular surveillance. In addition, our study highlights that more than a decade of bacterial genomic surveillance missed at least one-third of all AMR transmission events due to plasmids.

**IMPORTANCE:**

Antimicrobial resistance (AMR) poses a significant threat to human health. Most AMR determinants are encoded extra-chromosomally on plasmids. Although current infection control strategies primarily focus on clonal transmission of multidrug-resistant bacteria, until today, AMR plasmid transmission routes are neither understood nor analyzed in the hospital setting. In our study, we simultaneously determined both clonal, that is, based on chromosomes, and AMR plasmid transmissions during routine molecular surveillance by combining long-read sequencing with a novel real-time applicable software tool and validated all potential transmission events with epidemiological data. Our analysis determined not only the yet unknown plasmid transmissions within healthcare facilities or within the community but also resulted, in addition to the clonal transmissions, in at least a third more transmissions due to AMR plasmids.

## INTRODUCTION

The rise of antimicrobial resistance (AMR) poses a serious problem to healthcare systems worldwide (1). Many severe infections associated with multidrug-resistant bacteria (MDRB) are caused by gram-negative rods (2). In response to the growing threat of AMR, the World Health Organization has categorized several bacterial species as critical priority pathogens (3, 4). This group of pathogens includes carbapenem-resistant and third-generation cephalosporin-resistant Enterobacterales as well as carbapenem-resistant *Acinetobacter baumannii*, which are frequently associated with nosocomial transmissions and cause difficult-to-treat infections.

Bacterial plasmids and other mobile genetic elements (MGEs) are well recognized as key drivers of AMR (5). They facilitate the horizontal transfer of AMR among bacteria, enabling rapid dissemination of resistance even across different species. However, due to technical challenges, continuous monitoring of the spread of AMR plasmids has been too complex and time-consuming for routine hospital surveillance. For this reason, AMR plasmid transmissions were regarded as the elusive “dark matter” of genomic surveillance in hospitals, and it is still unclear to which extent plasmid transmission contributes to the overall burden of AMR. Recent improvements in long-read whole genome sequencing (lrWGS) made it possible to overcome many technical difficulties associated with previous sequencing technologies, such as the possible misassembly of repetitive sequences, which are frequent in MGE. Now, lrWGS enables not only a more accurate reconstruction of plasmids and other MGEs but also provides the basis for subsequent comparative analysis of plasmids to elucidate potential transmissions.

Although recent publications have already characterized AMR plasmids in the healthcare system, these studies only sequenced selected isolates by lrWGS and relied mainly on short-read WGS data to reconstruct plasmids (6–9). Additionally, most studies concentrated only on single resistance genes (10–15) and defined transmission events only by genomic data lacking epidemiological evaluation (16, 17). In contrast, the aim of this study was to continuously monitor AMR plasmids of all gram-negative MDRB in our hospital. We included both carbapenem- and beta-lactam-resistant MDRB, as ESBL/AmpC-producers are the most common gram-negative MDRB type at our hospital and German hospitals in general (3, 4).

For more than a decade, our infection control strategy already included prospective, that is, continuous and in real time, core genome multi-locus sequence typing (cgMLST) to identify transmissions of MDRBs (i.e., “clonal” transmissions based on chromosomal markers) (18). Now, after transitioning from short-read WGS to lrWGS and implementation of novel software tools to detect plasmid transmissions in real time, we assessed the burden of plasmid transmissions for our hospital compared with clonal transmissions and validated our findings with respect to putative transmission routes, that is, the transfer of plasmids within the host or between different patients, using epidemiological data. Taken together, we present a strategy to monitor plasmid epidemiology during clinical routine surveillance. This enabled us, in addition to the detection of clonal transmissions, to uncover plasmid transmission routes, thereby enlightening the dark matter of bacterial genomic surveillance and ultimately improving infection control practices to fight the spread of AMR in hospitals.

## MATERIALS AND METHODS

### Clinical setting and sample collection

The University Hospital Münster (UHM) is a 1,450-bed tertiary care center admitting 55,000 patients per year in Münster, Germany. All samples were prospectively collected between January 2022 and June 2023. The isolates (*n* = 540) enrolled in the study matched the definition for gram-negative MDRB as outlined below and originated from clinical samples as well as from screening efforts in high-risk areas or were part of outbreak investigations. As per German national infection control guidelines (19), MDRB were defined as either carbapenem-resistant organisms and/or ESBL/AmpC producers with quinolone resistance (adult patients) or ESBL/AmpC producers (neonatal intensive care patients). Inclusion of isolates was based on the antimicrobial susceptibility phenotype, which was performed on all isolates prior to sequencing. One sample per patient was included unless several MDRB species were detected in one patient or MDRB isolates of a single species showed different phenotypes.

### Microbiology methods: bacterial culture and identification

All collected isolates were cultivated and identified according to standardized microbiology techniques. Bacterial cultures from all specimens were performed using the respective selective media (chromID ESBL agar, bioMérieux, Marcy l’Étoile, France) or universal nutrient media (Columbia agar with 5% sheep blood, Becton Dickinson, Heidelberg, Germany; MacConkey, II agar, BD), depending on the sample origin, that is, screening vs. clinical specimen. Bacterial species were identified using matrix-assisted laser desorption/ionization time-of-flight mass spectrometry (MALDI-TOF/MS) (MALDI Microflex LT or MALDI Biotyper Sirius one IVD System, Bruker, Bremen, Germany) with identification scores above 2.0. Antimicrobial susceptibility testing was performed using a Vitek2 automated system (bioMérieux) applying EUCAST clinical breakpoints at the year of isolation. Carbapenem resistance was confirmed with the gradient diffusion method (Etest, bioMérieux).

### DNA extraction and long-read whole genome sequencing

For immediate sequencing irrespective of any suspicion of an outbreak, all MDRB isolates were selected based on their antimicrobial resistance phenotype. Genomic DNA of MDRB was extracted using either the Monarch Genomic DNA Purification Kit (New England Biolabs, Ipswich, MA, USA) or the ZymoBIOMICS 96 MagBead DNA Kit with Lysis tubes (Zymo Research, Freiburg, Germany) and sequenced on a PacBio Sequel IIe system using the SMRTbell Express Template Prep Kit 2.0 (Pacific Biosciences Inc., Menlo Park, CA, USA). Sequencing data were assembled *de novo* using the SMRT Link software suite 10 or 11 (Pacific Biosciences Inc.) with default parameters. An additional script determined the returned coverage and included information concerning circularity per contig into the FASTA headers of the consensus sequence FASTA file. Samples were excluded from subsequent analysis after lrWGS if the isolate (i) did not match species identified by microbiology report, (ii) failed during contamination check, (iii) contained only incomplete sequencing data illustrated by a deviation >10% of the expected genome size, or (iv) had an overall low sequencing quality, for example, due to base methylation-related homopolymer errors.

### Genotypic characterization of chromosomes and plasmids

All bioinformatic analyses were performed using SeqSphere^+^ software version v10 beta (Ridom GmbH, Münster, Germany) with NCBI AMRFinderPlus v3.11.26 (20, 21) and the long-read data plasmid transmission analysis module (22). Details of the analyses of chromosomes and plasmids are given in the following sections.

### Identification of potential transmissions by cgMLST

Standardized cgMLST schemes (https://www.cgmlst.org) (23), which represent chromosomal markers of the bacterial species, were used to compare *Acinetobacter baumannii*, *Escherichia coli*, *Klebsiella pneumoniae*, and *Serratia marcescens*. For all other species, in-house *ad-hoc* cgMLST schemes were defined. Isolates with a pairwise cgMLST allelic distance ≤5 alleles were identified as a potential transmission. Multilocus sequence typing (MLST) sequence types (STs) were extracted from the WGS data to provide additional genotypic information about the circulating lineages. Phylogenetic trees were constructed based on cgMLST using a neighbor-joining approach and were visualized with the web tool iTOL v. 6.8.1 (24).

### Plasmid characterization, identification of potential plasmid transmissions, and visualization

For plasmid reconstruction and subsequent typing, different modules from the MOB-suite v3.1.8 software (25) implemented in SeqSphere^+^ were used. Circular contigs shorter than 500 kb were automatically regarded as plasmids; contigs of fragmented plasmids were reconstructed using the MOB-recon module. Both circular contigs and reconstructed plasmids were analyzed with MOB-typer to predict replicon family, relaxase type, and mobility. The results of the MOB-suite were combined with NCBI AMRFinderPlus (20) and CGE MobileElement Finder v1.1.2 (26) results to localize AMR genes and integrative mobile genetic elements (iMGE) on the reconstructed plasmids, respectively. iMGEs were detected using a minimum nucleotide identity of 90% and a sequence alignment of 95%. To identify potential plasmid transmissions, Mash (v2.1) distances (27) between all priority AMR-carrying plasmids were calculated. Priority AMR genes were defined as genes coding for carbapenemases, extended-spectrum beta-lactamases, and/or class C beta-lactamases corresponding to WHO priority pathogens (4) and genes conferring resistance to colistin. Based on plasmid sequences, we constructed a Mash database with a k-mer size of 21 nucleotides and a sketch size of 10,000. For distance calculation, we additionally included a size correction, where for each 1% size difference of the plasmids, the corrected Mash distance was lowered by 0.0003 to compensate for insertions or deletions of larger repetitive sequence fragments. For plasmids with a size difference of more than 40%, the uncorrected value was kept to account for possible multimer formations. Plasmids with a (corrected) Mash distance of ≤0.001 were regarded as highly similar, suggesting a potential transmission (22). For a selection of plasmids with pairwise Mash distances below the threshold, a Quast v5.2 analysis with default parameters (28) was performed to evaluate additional genetic changes. Plasmids were annotated using Bakta Web (29), and comparisons were visualized by pyGenomeViz v0.4.4 that utilized MUMmer v3.23 for alignment (30).

### Epidemiological evaluation and validation of potential transmissions

All potential transmission events were counted as a single event between two isolates. Connected transmissions were summarized in clusters; therefore, the number of transmission events is the number of isolates per cluster without the index sample. Plasmid transmission events were counted as a single event between two isolates regardless of whether more than one plasmid was (co-)transferred. Genetically similar plasmids that occurred during clonal transmission events were not rated as plasmid transmission events. To evaluate potential priority AMR plasmid and/or clonal transmissions, patients′ medical records were reviewed for up to 1 year before sample collection to identify possible contacts between patients during previous hospital stays. To confirm potential transmissions, a hospital contact suggesting a healthcare-associated transmission was defined as a contact on room, ward, or department level. If the patient was admitted for at least one overlapping calendar day within the hospital stay to the same room or ward, it was categorized as “probable transmission.” An admission with contact on the department level was rated as “contact [healthcare associated],” respectively. A community contact suggesting a transmission outside the hospital was defined as a contact on the household level, sharing the same zip code, or other evidence for community contact, for example, family members. Further details stating the contact between patients are listed in the supplemental material (Tables S3 and S4).

### Statistical analysis

All analyses to determine basic statistical values were performed using R v4.3.1 (R Core Team: https://www.r-project.org/).

## RESULTS

### Characterization of MDRB

**Fig 1:**
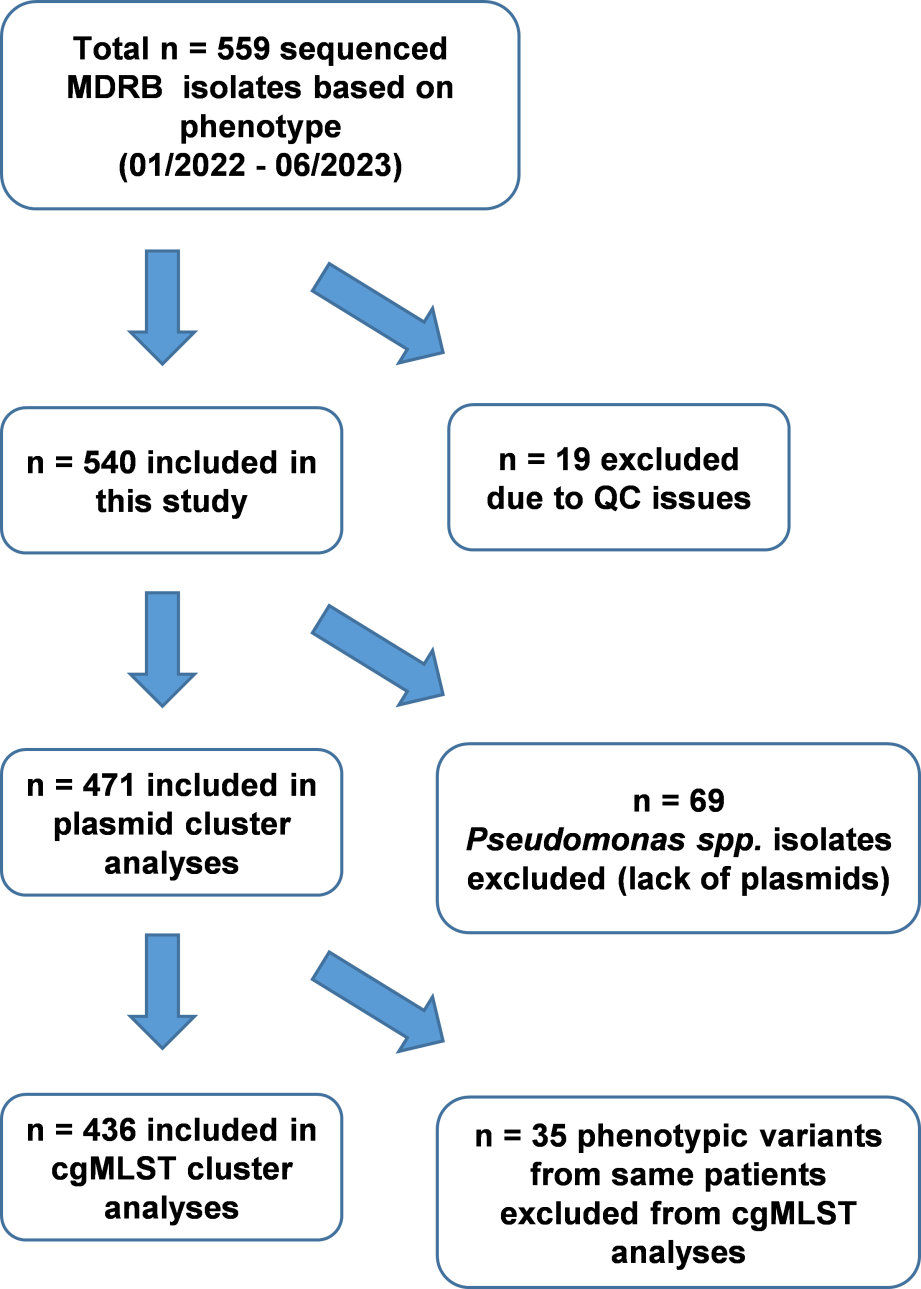
Flow chart of isolate inclusion. Number of isolates included in each analysis step and exclusion criteria. In total, 19 MDRB were excluded due to quality exclusion criteria: (i) species mismatched with microbiology report (*n* = 1), (ii) contamination check failed (*n* = 2), (iii) incomplete sequencing data (*n* = 4), and (iv) methylation-related homopolymer errors (*n* = 10) or low data quality (*n* = 2).

During the prospective surveillance of MDRB over a period of 18 months, we detected and characterized 540 isolates from 455 patients (Fig. 1; Table S1) using lrWGS. Of the 28 identified gram-negative species, *E. coli* (*n* = 278, 51.48%) and *K. pneumoniae* (*n* = 97, 17.96%) were the two most common (Table S2). The predominantly isolated strains were *E. coli* ST131 and *K. pneumoniae* ST307, respectively. The distribution of additional STs is displayed in Fig. S1. Within this data set, we detected carbapenemase genes in 27 isolates (2 *bla*_KPC-3_, 8 *bla*_NDM_, 14 *bla*_OXA_, and 3 *bla*_VIM_); nine encoded within the chromosome and 18 located on plasmids. In 499 isolates, ESBL-encoding genes were found, most frequently *bla*_CTX-M-15_ (197 isolates). Overall, priority AMR genes were encoded on the chromosome in 271 isolates. In 201 isolates, AMR genes were located on plasmids and chromosomes, and in 46 isolates, AMR genes were located on plasmids only. In 22 isolates, no priority AMR gene could be detected. Here, the antimicrobial resistance phenotype is most likely caused by other resistance mechanisms such as efflux pumps.

MOB-suite analysis detected a total of 1,539 plasmids in our lrWGS data set of 540 isolates. Of these, 1,299 plasmids were circular (84.41% of all plasmids). The 240 non-circular plasmids consisted of 378 non-circular contigs (104 single linear contigs and 136 multi-contig plasmids with 2–14 contigs) (Table S1). The overall number of plasmids per isolate varied between 0 and 13 (median 2, IQR 1–4) (Fig. S2) and ranged in size between 1.4 kb and 431.7 kb (Fig. S2). Of the 540 MDRB, 247 (45.7%) harbored at least one priority AMR plasmid with a maximum of three priority AMR plasmids per isolate in six isolates and two priority AMR plasmids in 25 isolates, respectively, with 237 (83.5%) of these being circular. For subsequent transmission analysis, we excluded the genus *Pseudomonas* because these isolates harbored almost no plasmids (Fig. S2). In total, 471 MDRB with 284 priority AMR plasmids from 405 patients were included in the final evaluation.

### Evaluation of clonal transmissions

Prior to the identification of potential clonal transmissions using species-specific cgMLST schemes, we excluded 35 additional isolates from the same patient with different phenotypes due to identical genotypes. Among the remaining 436 MDRB isolates, we identified 38 potential transmissions that were summarized to 24 potential clonal transmission clusters, that is, groups of genetically related isolates, among four different bacterial species involving 62 (14.2%) isolates (Table 1; Table S3). These clusters were subsequently evaluated based on patients' medical records. In seven of 24 potential clonal transmission clusters (cgMLST clusters 1, 5, 7, 11, 12, 16, and 20) comprising in total 19 clonal transmission events, epidemiological links were identified, resulting in the classification as a probable clonal transmission (Table 1; Table S3).

**TABLE 1:** Epidemiological evaluation of potential clonal transmission clusters^*a*^

cgMLST cluster #	Species (# samples)	ST	Maximal allelic distance	Epidemiological evaluation of transmissions
1	*Enterobacter hormaechei* (7)	104	1	Probable transmission
2	*E. hormaechei* (3)	190	5	Contact (healthcare associated)
3	*E. hormaechei* (2)	98	3	Contact (healthcare associated)
4	*Escherichia coli* (3)	69	5	No contact identified
5	*E. coli* (2)	131	3	Probable transmission
6	*E. coli* (2)	6396	3	No contact identified
7	*E. coli* (2)	131	1	Probable transmission
8	*E. coli* (2)	69	3	Contact (community associated)
9	*E. coli* (2)	69	5	Contact (healthcare associated)
10	*E. coli* (2)	1406	2	No contact identified
11	*E. coli* (2)	1193	1	Probable transmission
12	*E. coli* (2)	131	2	Probable transmission
13	*E. coli* (2)	131	5	No contact identified
14	*E. coli* (2)	131	2	Contact (healthcare associated)
15	*E. coli* (2)	131	5	Contact (healthcare associated)
16	*Klebsiella pneumoniae* (8)	3172	2	Probable transmission
17	*K. pneumoniae* (2)	661	2	Contact (healthcare associated)
18	*K. pneumoniae* (2)	307	2	Contact (healthcare associated)
19	*K. pneumoniae* (2)	15	0	No contact identified
20	*K. pneumoniae* (3)	12	1	Probable transmission
21	*K. pneumoniae* (2)	307	2	No contact identified
22	*K. pneumoniae* (2)	35	0	No contact identified
23	*K. pneumoniae* (2)	147	0	Contact (healthcare associated)
24	*Serratia marcescens* (2)	NA	0	Contact (healthcare associated)

^*a*^ cgMLST = core genome multilocus sequence typing. ST = MLST sequence type. NA = not applicable.

### Evaluation of priority AMR plasmid transmissions

**Fig 2:**
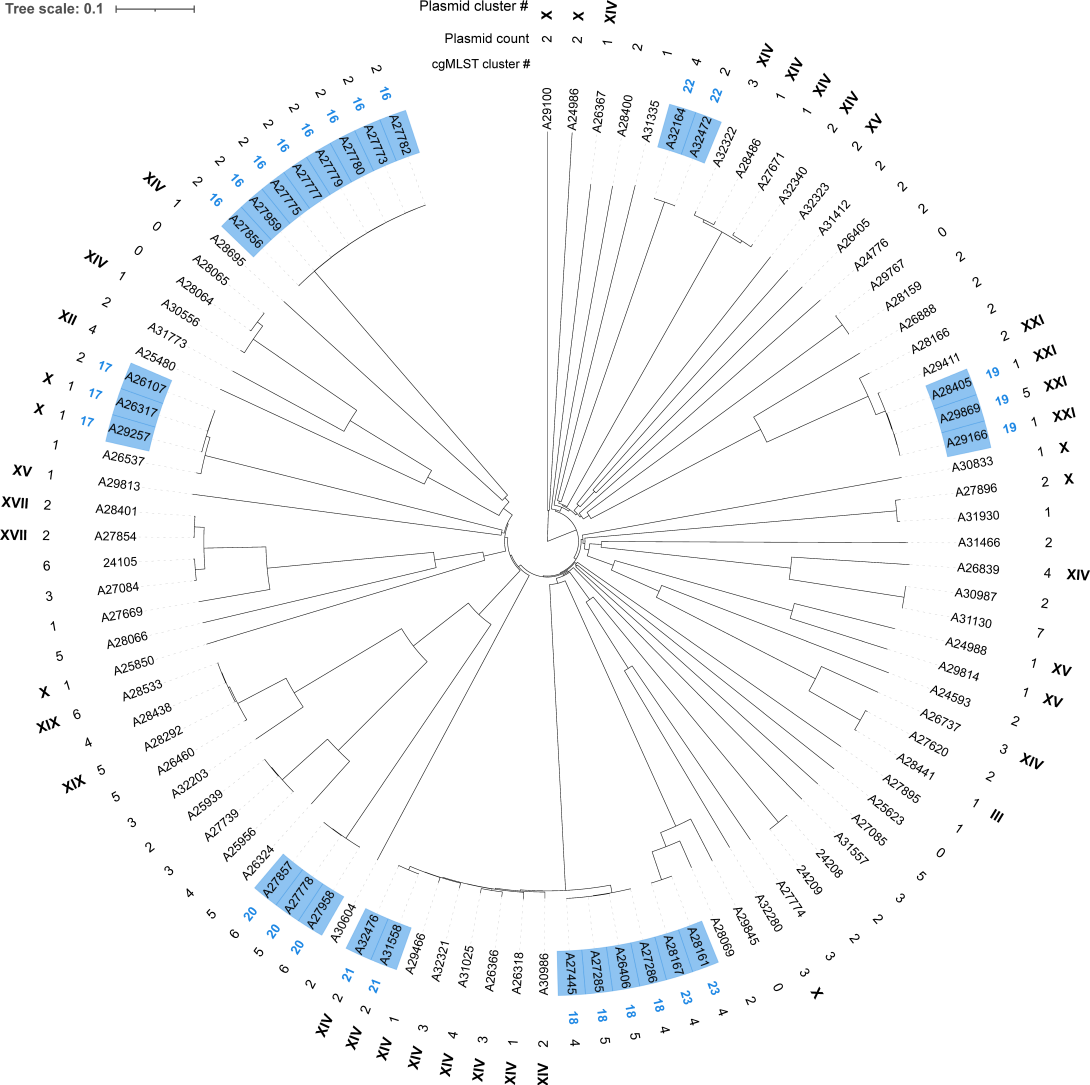
Phylogenetic tree of all 97 *Klebsiella pneumoniae* isolates including information on plasmid clusters. The tree is generated based on cgMLST allelic distances using a neighbor-joining algorithm. Isolate IDs of cgMLST clusters are colored in blue and numbered with Arabic numbers in blue (inner ring “cgMLST cluster #”) according to Table 1. The plasmid count per isolate is given in the middle ring, and plasmid clusters are named with Roman numbers (outer ring “Plasmid cluster #”) according to Table 2.

Next, we used Mash to identify potential priority AMR plasmid clusters. Mash is a rapid approach to compare sequences by using k-mers of nucleotides that also take the mosaic structure of plasmids into account. Among the 247 MDRB isolates that harbored at least one priority AMR plasmid, we identified 140 potential plasmid transmissions with highly similar plasmids (i.e., Mash distances ≤ 0.001) in 10 different species that were summarized to 37 potential plasmid clusters (Tables S4 and S5). Of these, 19 potential plasmid transmissions within thirteen plasmid clusters (Table S5) were excluded because they were already detected within clonal transmission clusters. The remaining 121 potential plasmid transmissions within 24 plasmid clusters (Table 2) were either within a single species with identical ST (plasmid clusters II, VIII; XX, XXI, and XIV) or with mixed STs (plasmid clusters I, V, VI, VII, XI, XIII, XIV, XVIII, XXII, and XXIII). In addition, there were nine plasmid clusters (plasmid clusters III, IV, IX, X, XII, XV, XVI, XVII, and XIX) containing multiple species. In total, 30.4% (143/471) of the analyzed isolates and 57.9% (143/247) of the isolates containing a priority AMR plasmid were assigned to a potential plasmid cluster. Frequent MDRB species *E. coli* and *K. pneumoniae* were found in 17 and eight plasmid clusters, respectively (Fig. 2; Fig. S4).

**TABLE 2:** Epidemiological evaluation of potential plasmid transmission clusters^*a*^

Plasmid cluster #	Species (# samples)	ST	Priority AMR gene	Epidemiological evaluation of transmissions (# transmissions)
I	*Escherichia coli* (3)	58/744	*bla*_SHV-12_	No contact identified
II	*E. coli* (4)	69	*bla*_CTX-M-1_	No contact identified
III	*Citrobacter freundii* (1), *Klebsiella pneumoniae* (1)	22, 441	*bla*_DHA-1_	No contact identified
IV	*E. coli* (17), *Klebsiella variicola* (1)	10/38/46/69/95/131/167, 925	*bla*_CTX-M-15/231_	Probable transmission (1) contact (community/healthcare associated)
V	*E. coli* (2)	10/381	*bla*_CTX-M-15_	No contact identified
VI	*E. coli* (5)	46/88/394/609	*bla*_CTX-M-3/15_	Probable transmission (1) and no contact identified
VII	*E. coli* (3)	10/69/88	*bla*_CTX-M-1_	Contact (healthcare associated)
VIII	*E. coli* (2)	453	*bla*_CTX-M-14_	No contact identified
IX	*Enterobacter hormaechei* (1), *E. coli* (6)	97, 88/770/1406/8371/15441	*bla*_SHV-12_	Probable transmission (1) and contact (healthcare associated)
X	*K. pneumoniae* (6), *Klebsiella quasipneumoniae* (2)	36/182/469/661/2010/6860, 2059	*bla*_CTX-M-15_	Contact (healthcare associated)
XI	*E. coli* (4)	10/58	*bla*_CTX-M-15_	Contact (healthcare associated)
XII	*E. coli* (1), *K. pneumoniae* (1)	95, 353	*bla*_SHV-12_	Probable transmission (1)
XIII	*E. coli* (2)	10/69	*bla*_DHA-1_	No contact identified
XIV	*K. pneumoniae* (17)	29/37/307/405/556/584/4452	*bla*_CTX-M-15_	Contact (healthcare associated)
XV	*E. coli* (1), *K. pneumoniae* (4)	1081, 13/17/462	*bla*_CTX-M-14_	Probable transmission (1)
XVI	*Citrobacter farmeri* (1), *Klebsiella michiganensis* (2)	852, 610	*bla*_CTX-M-206_	Probable transmission (1)
XVII	*Klebsiella oxytoca* (2), *K. pneumoniae* (2)	199, 45	*bla*_CTX-M-15_	Probable transmission (1)
XVIII	*E. coli* (3)	69/224	*bla*_CTX-M-65_	No contact identified
XIX	*K. pneumoniae* (2), *Morganella* *morganii* (1)	11, NA	*bla*_OXA-48_	Contact (healthcare associated)
XX	*E. coli* (2)	1193	*bla*_CTX-M-27_	No contact identified
XXI	*K. pneumoniae* (4)	15	*bla*_CTX-M-15_	Contact (healthcare associated)
XXII	*E. coli* (38)	10/131/10220/15440	*bla*_CTX-M-15/27/231_	Probable transmission (5) contact (healthcare associated)
XXIII	*E. coli* (2)	131/1011	*bla*_CTX-M-15_	No contact identified
XXIV	*E. coli* (2)	1193	*bla*_CTX-M-15_	Contact (healthcare associated)

^*a*^ ST = MLST sequence type. AMR = antimicrobial resistance. NA = not applicable.

Many plasmid clusters (*n* = 17) contained only two to four isolates; the clusters IV, XIV, and XII contained 18, 17, and 38 isolates, respectively. Interestingly, these three large plasmid clusters all contained *bla*_CTX-M-15_ as the target gene. Further details on the plasmid clusters are listed in Tables S2 and S4.

Subsequently, the 24 plasmid clusters were evaluated based on epidemiological information using the same epidemiological evaluation criteria as for the clonal transmission clusters (Table 2; Table S4). In eight of the 24 plasmid clusters (plasmid clusters (IV, VI, IX, XII, XV, XVI, XVII, and XXII), we confirmed 12 probable plasmid transmissions within the plasmid clusters based on epidemiological information (Table 2). Of these, we detected seven cases of transmission within the same patient (intra-host transmission) and five plasmid transmissions between different patients with evidence of contact (Table S4).

### Description of a *bla*_CTX-M-15_-carrying plasmid with evidence of clonal and plasmid-based transmission

**Fig 3:**
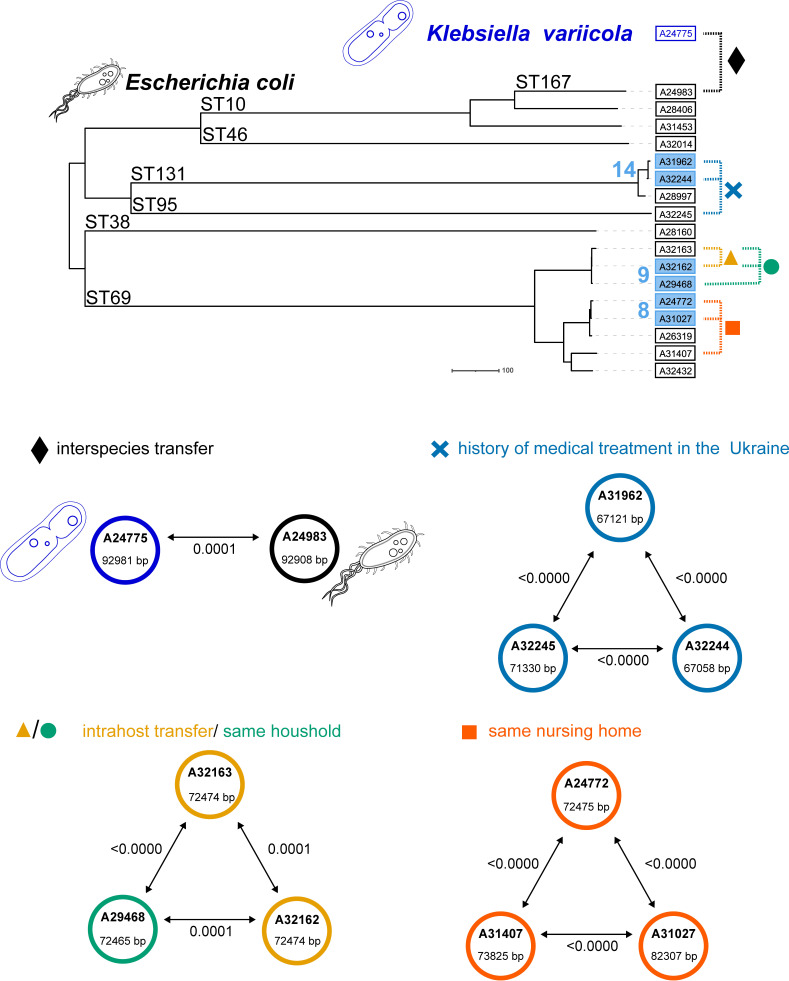
Transmission events in plasmid cluster IV. The phylogenetic tree of *Escherichia coli* is based on cgMLST distances constructed using a neighbor-joining algorithm. Identified clonal clusters are given in blue Arabic numbers, and different plasmid transmission events as well as epidemiological links are indicated by dashed, colored lines (colored symbols). This example illustrates both interspecies plasmid transfer (black rhombus) and intra-host plasmid transfer (yellow triangle). In addition, there is evidence for clonal or plasmid transmissions based on patients living in the same household (green dot), residing in the same nursing home (red square), or sharing a history of medical treatment in Ukraine (blue cross). Comparisons of plasmids and the plasmid sizes in base pairs (bp) are given inside the circles, the pairwise Mash distances are illustrated along the arrows and are detailed in Table S6.

One example illustrating several different modes of transmission within the hospital environment and community setting is plasmid cluster IV comprising 18 MDRB (Fig. 3; Table 2). This cluster is characterized by a genetically highly similar *bla*_CTX-M-15_-carrying plasmid associated with *E. coli* (*n* = 17). The majority of isolates are classified as ST69, but the plasmid cluster also spans across seven different *E. coli* STs and a single *Klebsiella variicola* isolate. All plasmids were characterized as IncFIA/IncFIC plasmids. A MOB_F_ relaxase was detected in 17 of 18 plasmids. As part of this cluster, three distinct clonal transmission chains were identified (cgMLST clusters 8, 9, and 14, Table 1; Fig. 3). Two of these clonal transmission clusters show potential community contact (same household or nursing home, cgMLST clusters 8 and 9). One clonal transmission cluster connects patients with a history of medical treatment in Ukraine (cgMLST cluster 14).

Our lrWGS approach enabled us to analyze the plasmids in more depth, including point mutations or duplications of resistance genes (Table S7; Fig. S5 through S8). Interestingly, although the plasmids of plasmid cluster IV were overall similar, the isolate A31453 harbored a different AMR resistance gene (*bla*_CTX-M-231_), which differed in a single, non-synonymous point mutation (E31A). Furthermore, in isolate A31027, we found an additional gene copy of *bla*_CTX-M-15_, which could potentially impact the antibiotic resistance phenotype of this isolate.

The plasmid with the lowest Mash distance, that is, the most similar plasmid to the one detected in *K. variicola* A24775, was found in the *E. coli* isolate A24983 with a Mash distance of 0.0001. Here, no epidemiological link could be confirmed, but we rate this as an example of inter-species horizontal gene transfer (Fig. 3) at some point in the past. Furthermore, the *E. coli* isolates A32162 and A32163, collected from the same patient at the same time point, share a plasmid with a Mash distance of 0.0001. This can be regarded as a case of intra-host plasmid transmission (Fig. 3). Interestingly, lower Mash distances (< 0.0001) were found for A32163 in comparison to A29468 and A24772, indicating that these plasmids are genetically very similar, although they are harbored in different bacterial isolates. In three other cases, we found evidence of transmission events outside of our hospital, referred to as community or healthcare-associated contacts (Fig. 3). Here, patients shared the same household, resided in the same nursing home, or had received medical treatment in Ukraine previously. iMGEs harboring the *bla*_CTX-M_ gene in the isolates of this plasmid cluster were nested in their plasmids and could not be detected in other isolates of our data set. Therefore, no evidence for the transposition of iMGEs was found.

## DISCUSSION

The aim of our study was to elucidate the dissemination of AMR plasmids in the hospital setting. We have been collecting WGS-based molecular surveillance data for more than a decade at our hospital to detect nosocomial clonal transmission events (18). After changing sequencing methods to lrWGS in 2021, we started to integrate the information on AMR-carrying plasmids into our routine surveillance. To evaluate this procedure, we analyzed 540 MDRB isolates collected over an 18-month period. In order to achieve a broad and unbiased overview of plasmid-mediated AMR transmissions, we included all gram-negative bacterial species rather than focusing on a particular resistance mechanism. Combining a Mash-based approach for plasmid comparisons with epidemiological contact tracing, we were able to identify 12 plasmid transmissions in eight probable plasmid clusters within this period in addition to the seven probable clonal transmission clusters consisting of 19 clonal transmissions (Tables 1 and 2). This suggests that AMR plasmid transmissions are less frequent in our hospital than clonal transmissions, resulting in at least one-third additional transmission clusters that have been missed for more than a decade during genomic surveillance. The transition to lrWGS data analysis also further improved the in-depth characterization of AMR. For example, we could determine the mosaic structure of plasmids and detect duplication of AMR genes (Fig. 3; Fig. S7). In fact, these gene duplications contributed to increased antimicrobial resistance (31). Taken together, our study shows the advantages of lrWGS in combination with novel software tools for routine molecular surveillance to monitor the spread of AMR.

Although our sequencing facility uses a PacBio Sequel IIe system, Mash-based approaches for plasmid analysis have also been used on data sets of hybrid assemblies sequenced by Oxford Nanopore Technologies and Illumina (22, 32). Over the recent years, the use of lrWGS has become more common, and very recently, ONT improved significantly with respect to error rates (33). As ONT requires relatively low capital investment, it is especially attractive for small and medium-sized laboratories. lrWGS provides several advantages compared with short-read sequencing technologies, as outlined above. Healthcare facilities that use short-read sequencing technologies to follow up on transmission pathways should be aware of the limitations of the technology and use lrWGS when plasmid transmissions are suspected.

Recent studies characterizing plasmids in the healthcare setting have focused on carbapenem-resistant bacteria or a specific carbapenemase (34–36). Naturally, this choice affects the plasmidome of the data set. It is likely that not all AMR-carrying plasmids are equally efficient in disseminating within the bacterial population. Hence, three categories to characterize the mobility of plasmids have been established based on the gene content of the plasmid, that is, conjugative, mobilizable, or non-mobilizable (25). A study on publicly available sequencing data of *Salmonella* could show the varying distribution of the plasmidomes and dissemination of AMR plasmids (37). Hence, the MDRB study population and its plasmidome will likely influence the rate of plasmid-mediated transmissions. This might explain differing estimates for transmission rates among different studies (35). In our study, most AMR plasmid clusters only contained a few isolates; however, three clusters (IV, XIV, and XII) comprised between 17 and 38 isolates. The plasmids shared among these three plasmid clusters all contain the beta-lactamase gene *bla*_CTX-M-15_. It appears that the largest plasmid clusters are dominated by *E. coli* ST131. It seems likely that here the plasmid is well adapted within this *E. coli* lineage and is conjugative for further dissemination. This conclusion is also in line with previously published data by Reuland et al. (38). It is tempting to speculate that these plasmids are efficient in transferring AMR and contribute to the successful dissemination of *bla*_CTX-M-15_ in the bacterial population. To investigate this further, however, more detailed epidemiological studies are required.

Our study has some limitations. First, because currently, only very few hospitals use lrWGS prospectively, our data set is based on only a single center. Besides this technical reason, the characterization of the AMR genes present in our MDRB isolates indicated that the overall epidemiology in our data set is similar to other hospitals in Germany (39). Although the number of detected genes encoding carbapenemases was low (*n* = 27), the most common AMR gene in our MDRB isolates was *bla*_CTX-M-15_ (*n* = 197). Because of the overall low numbers of nosocomial transmission events in our study population, we were not able to perform a detailed statistical analysis of risk factors for plasmid transmission in the hospital setting. A recent study from a different healthcare setting with a much higher rate of transmissions is in line with our results and also showed a high contribution of plasmid-mediated transmissions (35). We therefore believe that our results are representative of countries with similar AMR epidemiology and corroborate with countries with a higher incidence of carbapenemase-producing Enterobacterales (35). The fact, however, that all studies including this study only count AMR-harboring plasmids (i.e., 284 of 1,539 plasmids detected in this study), results in an underestimation of the total number of plasmid transmissions. Second, our infection control policies and our healthcare setting influence our study. For example, although our hospital is not a single-room-only facility, most patients are cared for in two-bed cubicles. According to our German national guidelines (19), patients are only screened for MDRB in high-risk areas if risk factors for colonization are known or as part of outbreak investigations. Hence, some carriers of MDRB might not be detected during their admission. A third factor that influences our results is the assignment and evaluation of transmission events. From a technical perspective, the similarity thresholds to assign either clonal or plasmid transmission events are dependent on the chosen values. Starting from our previous investigations (18), we have chosen a more stringent allelic distance for clonal transmissions. To further improve plasmid comparisons, we added a size correction to better reflect the mosaic nature of plasmids in addition to the recently published Mash thresholds (37). Nevertheless, mobile/transposable elements carrying AMR genes (13) still might go undetected if the recipient structure differs significantly from the donor. Furthermore, it seems plausible that plasmid transmission is a “slow” process because, in addition to the transfer of the AMR plasmid-harboring organism, the AMR plasmid itself must be transferred to the receiving bacterial species, very likely as an intra-host process. Therefore, epidemiological evidence for plasmid transmissions might be harder to find in the hospital setting, especially as MDRB carriers are not routinely screened sequentially over longer time periods.

AMR plasmid transmission most likely starts within a patient (intra-host transmission) but can also be traced across several patients (inter-host transmission). Although our study uncovered examples of all of these types of AMR transmissions, the study was not designed to investigate the sequence of these events. However, based on our observations, we hypothesize that intra-host plasmid transmission is the first and likely the crucial step in this process. Here, AMR plasmids spread within a bacterial population, across STs and different species. From an infection prevention perspective, our data and interpretation highlight the importance of the rational use of antibiotics. Antibiotic stewardship guidelines will reduce selection pressure for AMR within individual patients but take time to be effective (40). Therefore, reducing plasmid-based AMR transmission in hospitals will be a long-term goal. In fact, a recent publication observed that the effects of early detection and isolation of colonized patients were more impactful in preventing clonal than plasmid-mediated transmissions. This could be explained by unrecognized reservoirs, such as environmental sources or ongoing “hidden” plasmid transmission outside the study setting (35). This is also in line with our observations that several transmission events had connections to other hospitals, abroad or local, nursing homes, or the community.

Our data demonstrate that the transmission of AMR is a multi-layered process. We were able to enlighten the dark matter of genomic surveillance in the healthcare setting using lrWGS combined with real-time applicable tools and emphasized the importance of molecular surveillance in understanding AMR dynamics. The finding that the detection of AMR plasmid transmissions significantly contributed to the total amount of AMR transmissions urges the need for targeted infection control measures. To contextualize local data and ultimately reduce AMR, regional and national networks and public health efforts are necessary to better understand and prevent the spread of AMR.

## Data Availability

lrWGS data are available at NCBI GenBank (https://www.ncbi.nlm.nih.gov/genbank/) under Bioproject number PRJNA1136676.

## SUPPLEMENTAL MATERIAL

Supplemental figures jcm.00121-25-s0001.docx Fig. S1 to S8.

Supplemental tables jcm.00121-25-s0002.xlsx Tables S1 to S7.

ASM does not own the copyrights to Supplemental Material that may be linked to, or accessed through, an article. The authors have granted ASM a non-exclusive, world-wide license to publish the Supplemental Material files. Please contact the corresponding author directly for reuse.
