# Supplementary material for: The dark matter of bacterial genomic surveillance—antimicrobial resistance plasmid transmissions in the hospital setting
Source: J Clin Microbiol. 2025 May 12;63(6):e00121-25. doi: 10.1128/jcm.00121-25 (PMC12153326; doi:10.1128/jcm.00121-25)
Supplement: Supplemental figures — Fig. S1 to S8. [file jcm.00121-25-s0001.docx]

**The dark matter of bacterial genomic surveillance - antimicrobial resistance plasmid transmissions in the hospital setting**

Annika Sobkowiak^1,2‡^, Vera Schwierzeck^1‡^, Vincent van Almsick^1,2‡^, Natalie Scherff^1^, Franziska Schuler^3^, Kyrylo Bessonov^4^, James Robertson^4^, Dag Harmsen^5*^, Alexander Mellmann^1*#^

**Supplementary figures**

Figure S1: Distribution of included multidrug-resistant species

Figure S2: Identified species and number of plasmids per species

Figure S3: Boxplot of the size of all plasmids

Figure S4: Phylogenetic tree of all 278 *Escherichia coli* isolates

Figure S5: Comparison of plasmids of plasmid cluster IV (interspecies transfer)

Figure S6: Comparison of plasmids of plasmid cluster IV (intra-host transfer/ same household)

Figure S7: Comparison of plasmids of plasmid cluster IV (same nursing home)

Figure S8: Comparison of plasmids of plasmid cluster IV (history of medical treatment in the Ukraine)


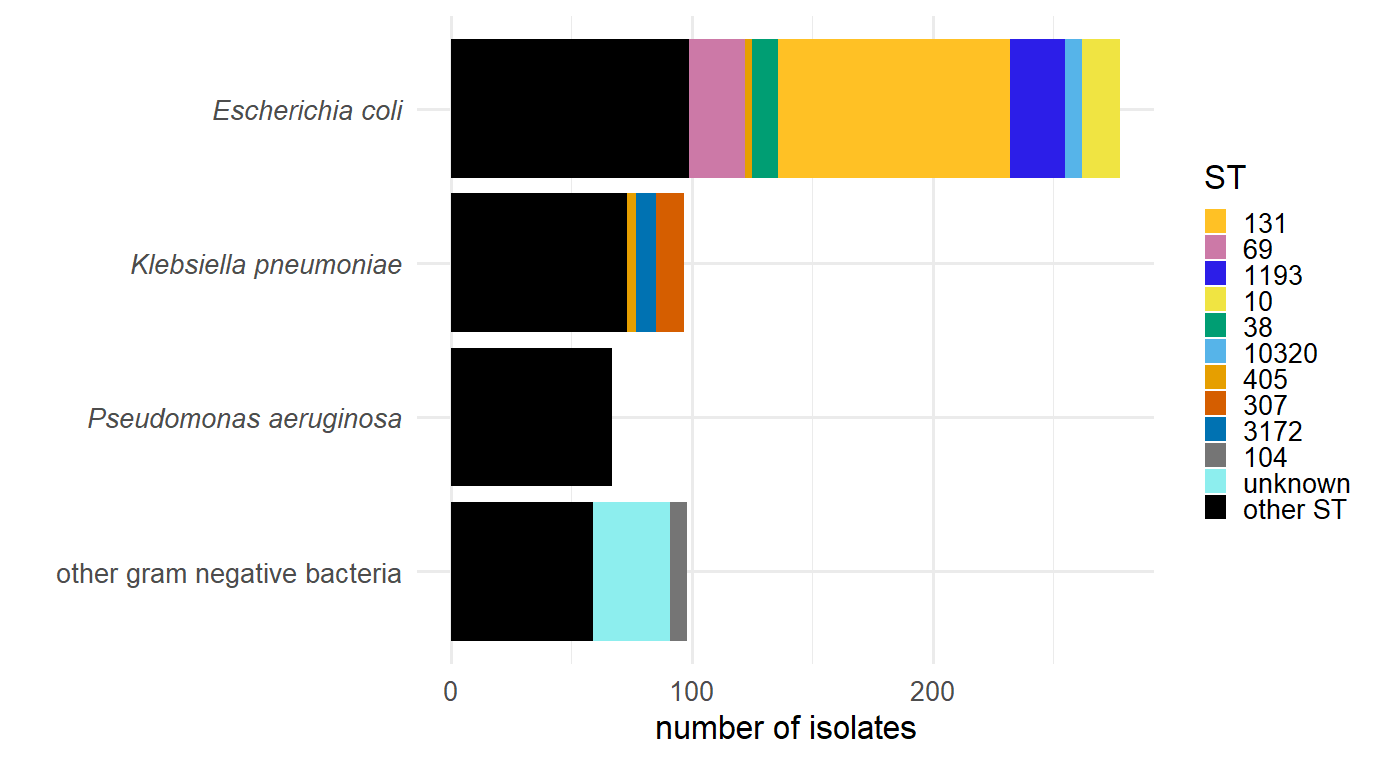


**Figure S1: Distribution of included multidrug-resistant species and frequent sequence types (ST)** (n=540)


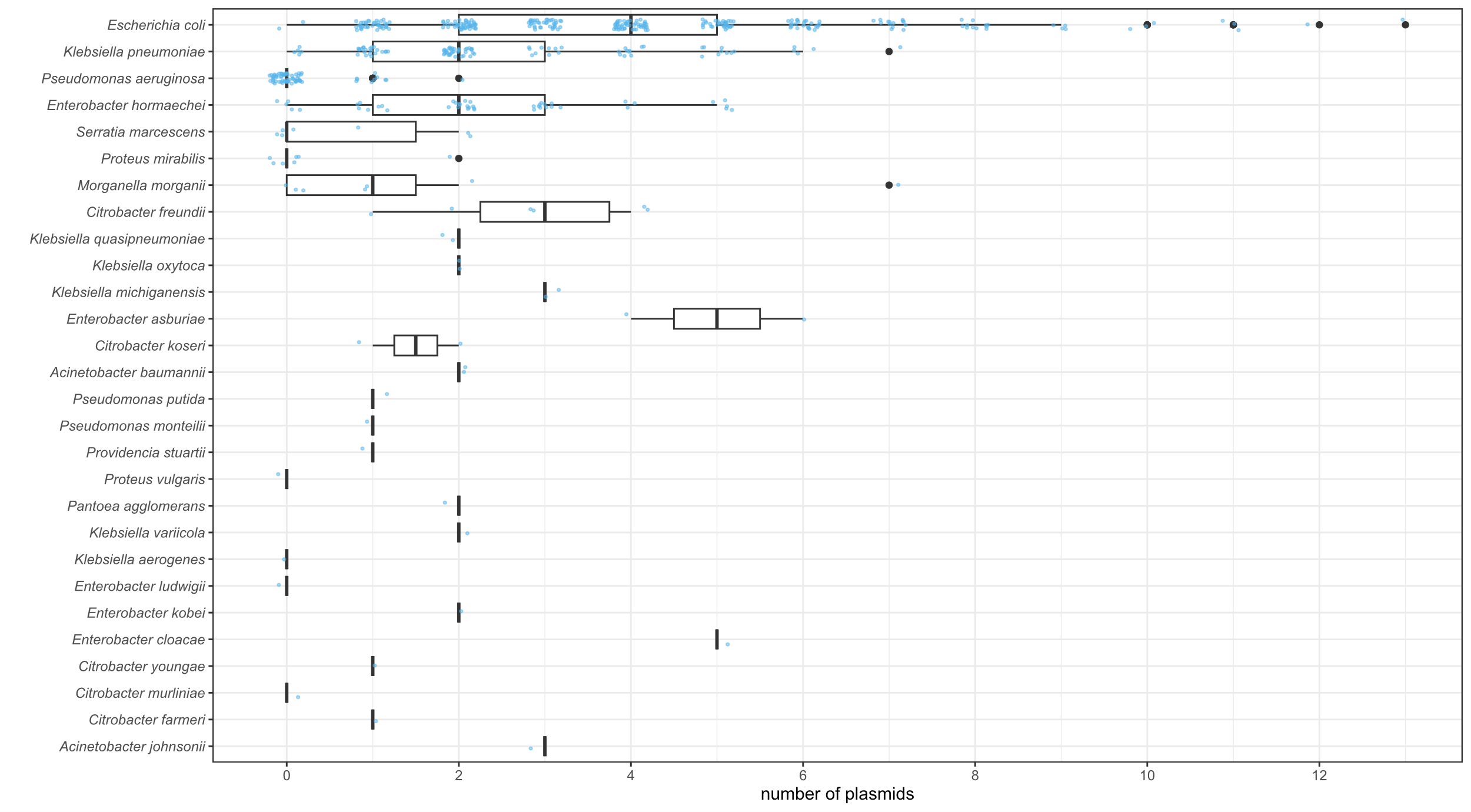


**Figure S2: Identified species and number of plasmids per species shown as box plots, each jitter represents one isolate of the dataset** (n = 540)

**
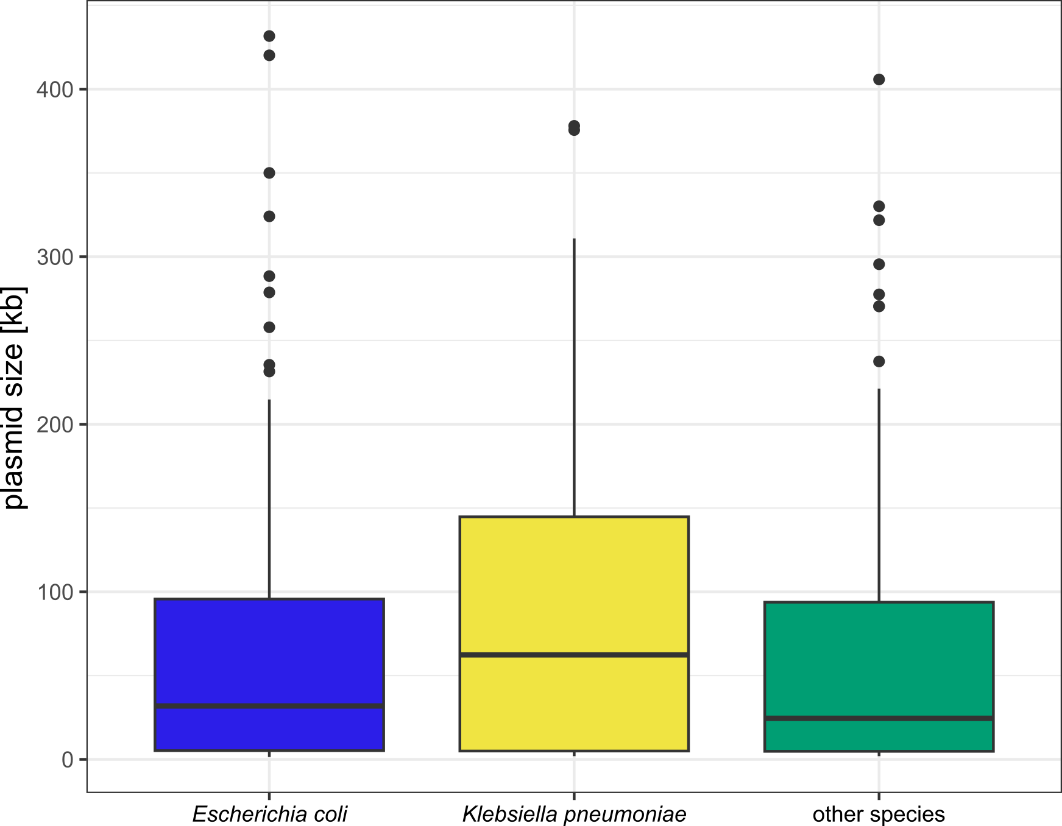
**

**Figure S3: Boxplot of the size of all plasmids, grouped by species**

**Figure S4: Phylogenetic tree of all 278 *Escherichia coli* isolates including information on plasmid clusters.** The tree is generated based on cgMLST allelic distances using a neighbor-joining algorithm, isolate IDs of cgMLST clusters are colored in blue (copy strains are included for illustration purposes) and numbered with Arabic numbers in blue (inner ring “cgMLST cluster #”) according to Table 1. The plasmid count per isolate is given in the middle ring and plasmid clusters are named with Roman numbers (outer ring “Plasmid cluster #”) according to Table 2.


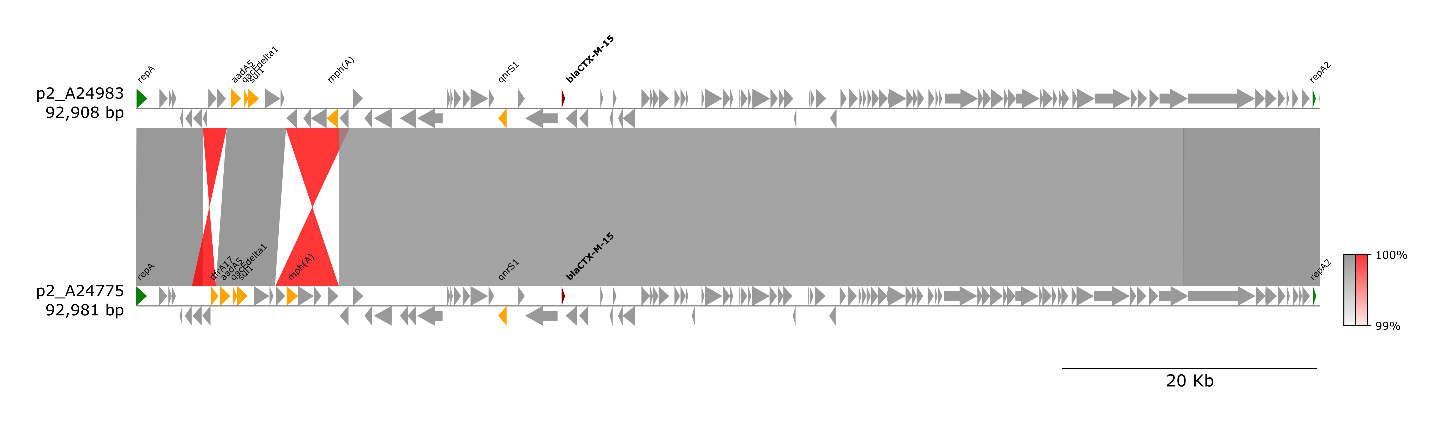


**Figure S5: Comparison of plasmids of cluster IV (interspecies transfer).** Comparison of the priority AMR plasmids detected in isolate A24775 and A24983 (interspecies transfer). Coding sequences (CDS) are plotted as arrows and selected features are labelled with gene names from Bakta annotation. Origins of replication are colored in green, the priority AMR gene in red with bold letters and other AMR genes in orange. Identical sequence regions are indicated as gray blocks and inverted regions as red.


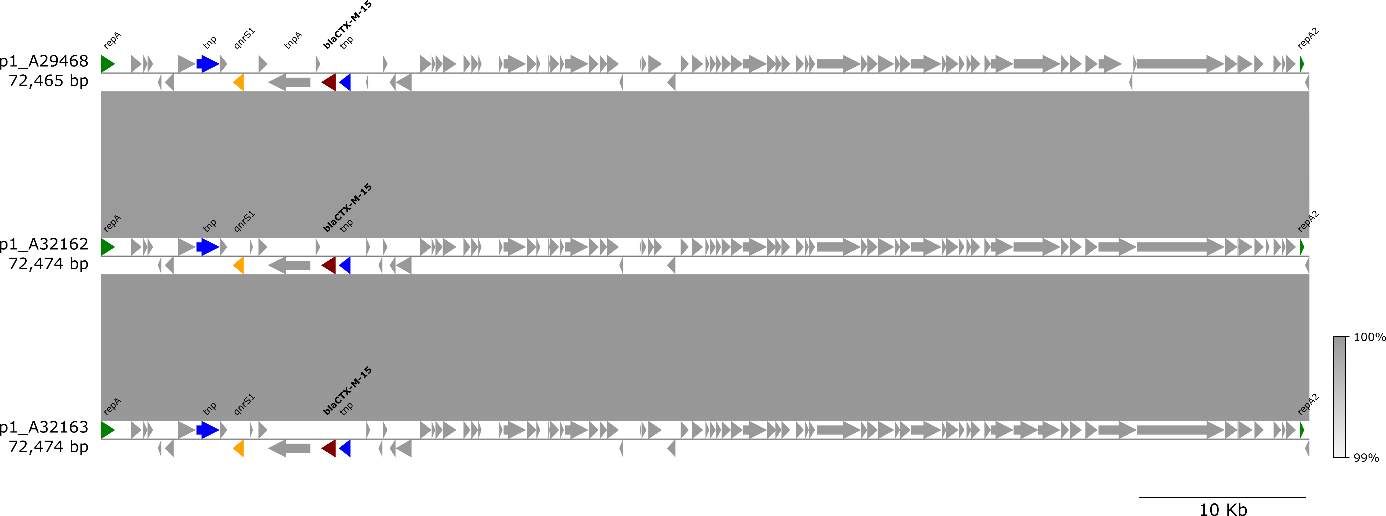


**Figure S6: Comparison of plasmids of plasmid cluster IV (intra-host transfer/ same household).** Comparison of the priority AMR plasmids detected in isolate A32163, A29468 and A32162 (intra-host transfer/ same household). Coding sequences (CDS) are plotted as arrows and selected features are labelled with gene names from Bakta annotation. Origins of replication are colored in green, the priority AMR gene in red with bold letters and other AMR genes in orange, flanking IS elements of composite transposons are colored in blue. Identical sequence regions are indicated as gray blocks.


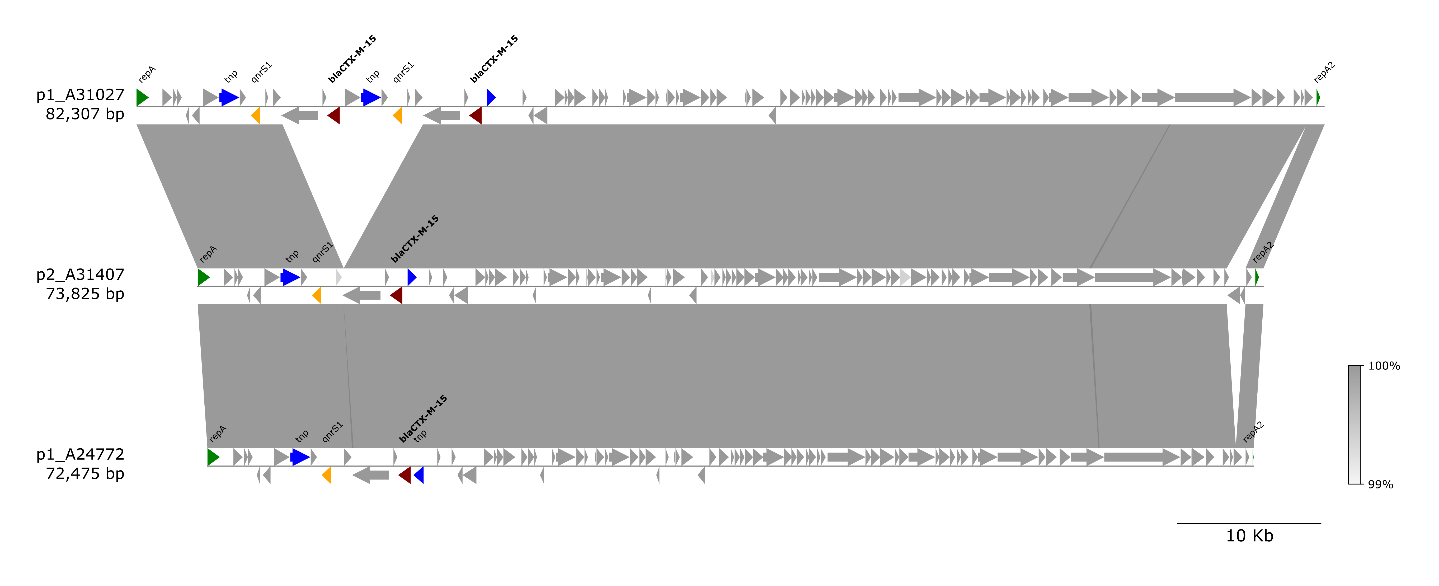


**Figure S7: Comparison of plasmids of plasmid cluster IV (same nursing home).** Comparison of the priority AMR plasmids detected in isolate A24772, A31027 and A31407 (same nursing home). Coding sequences (CDS) are plotted as arrows and selected features are labelled with gene names from Bakta annotation. Origins of replication are colored in green, the priority AMR gene in red with bold letters and other AMR genes in orange, flanking IS elements of composite transposons are colored in blue. Identical sequence regions are indicated as gray blocks.


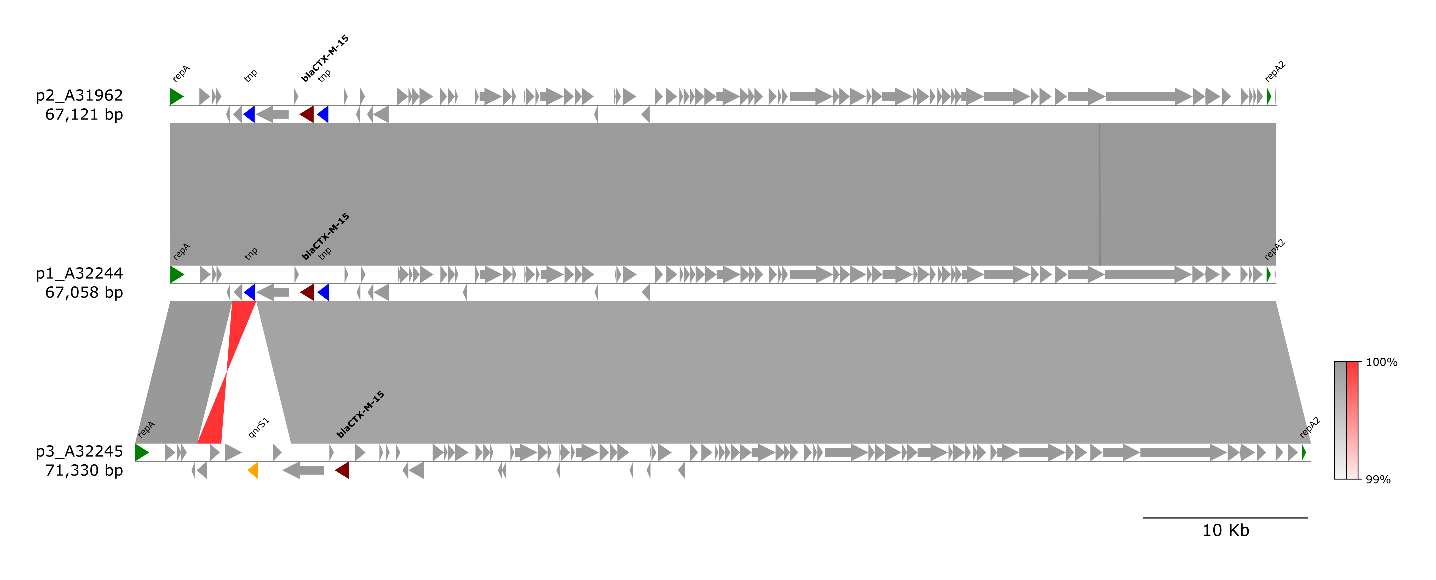


**Figure S8: Comparison of plasmids of plasmid cluster IV (history of medical treatment in the Ukraine).** Comparison of the priority AMR plasmids detected in isolate A32962, A32244 and A32245 (history of medical treatment in the Ukraine). Coding sequences (CDS) are plotted as arrows and selected features are labelled with gene names from Bakta annotation. Origins of replication are colored in green, the priority AMR gene in red with bold letters and other AMR genes in orange, flanking IS elements of composite transposons are colored in blue. Identical sequence regions are indicated as gray blocks and inverted regions as red.
